# The Impact of Smoking on Airflow Limitation in Subjects with History of Asthma and Inactive Tuberculosis

**DOI:** 10.1371/journal.pone.0125020

**Published:** 2015-04-27

**Authors:** Hyun Jung Kim, Seunghee Baek, Hee Jin Kim, Jae Seung Lee, Yeon-Mok Oh, Sang-Do Lee, Sei Won Lee

**Affiliations:** 1 Department of Pulmonary and Critical Care Medicine, and Clinical Research Center for Chronic Obstructive Airway Diseases, Asan Medical Center, University of Ulsan College of Medicine, Seoul, South Korea; 2 Department of Internal Medicine, Kyungpook National University School of Medicine, Daegu, South Korea; 3 Department of Clinical Epidemiology and Biostatistics, Asan Medical Center, University of Ulsan College of Medicine, Seoul, South Korea; 4 The Korean Institute of Tuberculosis, Osong, South Korea; University of Alabama at Birmingham, UNITED STATES

## Abstract

**Background:**

Although smoking is the most important and modifiable cause of chronic obstructive pulmonary disease (COPD), other risk factors including asthma and tuberculosis (TB) are also associated. It is common for COPD patients to have more than one of these risk factors. The aims of this study were to determine the prevalence of airflow limitation (FEV_1_/FVC<0.7) according to the risk factors and to investigate their impact and interaction in airflow limitation.

**Methods:**

From the Korean National Health and Nutrition Examination Survey between 2008 and 2012, we analyzed participants over 40 years of age by spirometry, chest radiograph and questionnaire about asthma and smoking history.

**Results:**

Of 12,631 participants, 1,548 (12.3%) had airflow limitation. The prevalence of airflow limitation in smokers (≥10 pack-year), asthmatics, and those with inactive TB was 23.9%, 32.1%, and 33.6%. The prevalence increased with the number of risk factors: 86.1% had airflow limitation if they had all three risk factors. Impacts of inactive TB and asthma on airflow limitation were equivalent to 47 and 69 pack-years of smoking, respectively. Airflow limitation resulted from lower levels of smoking in those with inactive TB and asthma. A potential interaction between smoking and inactive tuberculosis in the development of airflow limitation was identified (p = 0.054).

**Conclusions:**

Asthma and inactive TB lesions increase susceptibility to smoking in the development of airflow limitation. People with these risk factors should be seen as a major target population for anti-smoking campaigns to prevent COPD.

## Introduction

Chronic obstructive pulmonary disease (COPD) is a major global health problem, with a prevalence of 5 to 25% among adults worldwide[1]. COPD is among the ten most common chronic health conditions and causes a marked restriction of daily activities and substantial utilisation of healthcare resources[1,2]. Furthermore, COPD is a leading cause of morbidity and mortality worldwide, and is expected to become the third leading cause of death by the year 2020[2,3]. The economic cost of COPD in the US was estimated to reach $37.2 billion[4].

COPD is a preventable and treatable disease[5]. For prevention, management of risk factors is essential, among which quitting smoking is the most important[6,7]. However, about 15 to 50% of patients with COPD are non-smokers[8] and recent studies have suggested other factors strongly associated with COPD, including chronic asthma[9] and pulmonary infection, particularly pulmonary tuberculosis (TB)[10–12]. TB has been accepted as a risk factor for COPD[13]; even a minimal TB lesion can be a risk factor[12]. The clinical and physiologic features of asthma are initially distinct from COPD, but with time lung function declines and the airflow limitation become irreversible[9,14] which is one of the hallmark features of COPD. Therefore, asthma and COPD may develop quite similar physiologic features, as indicated in epidemiologic studies[9,15].

Smoking, TB and asthma are all known as factors associated with COPD. However, interactions between these associated factors in the development of airflow limitation have received little attention. In practice, many people have more than one of these associated factors simultaneously; e.g., smoking asthmatics or smokers with TB history. This study aimed to determine the prevalence of airflow limitation according to the major risk factors (inactive TB, asthma, and smoking) and to investigate potential interactions among them. Korea has an intermediate burden of TB, and asthma is also prevalent[16], making it a suitable country in which to undertake this study.

## Materials and Methods

### Participants and Data Collection

We analyzed data from the Korea National Health and Nutrition Examination Survey (KNHANES), conducted from 2008 to 2012. A stratified, multi-stage, clustered, probability sampling design was used to produce a nationally representative sample of the resident, civilian Korean population[17,18]. The participants, who were at least 40 years old, had a health care interview and basic medical examinations. Among possible participants, we enrolled those who had both acceptable spirometry data and information of asthma and smoking history. We excluded participants if they had another identifiable lung disease except inactive TB (e.g. active TB, pneumonia or tumor) on chest radiograph.

### Ethics Statement

The survey was approved by the institutional review board (IRB) of Korea Center for Disease Control and Prevention. Informed consent was obtained from all participants during the initial data collection.

### Questionnaires and Anthropometry

Trained interviewers administered the standardized questionnaire about chronic illness (e.g. hypertension, hyperlipidemia, angina, hemorrhoid, arthritis, rheumatic arthritis, bronchiectasis, depression, epilepsy, chronic kidney disease, diabetes mellitus, thyroid disease, cancer and hepatitis), health behavior(e.g. medical check-up, vaccination, daily activity, medical utilization, alcohol, smoking, exercise, suicide attempt, menstrual cycle and breast feeding) and socioeconomic status (e.g. property, residential types, income, education). Smoker was defined as someone who had smoked ≥100 cigarettes for lifetime in KNHANES. Meanwhile, in the analysis of smoking as the associated factor with airflow limitation, we defined smokers as the subjects who had above 10 pack-years smoking history, which is the significant risk factor of COPD[17,18]. The form of smoking was not specified, because other types of smoking than cigarette were scarce in Korea. Participants were categorized in terms of the presence or absence of asthma, which was defined by an affirmative response to the question, “Have you ever had asthma?” Chronic bronchitis was defined if the subjects had cough or sputum for more than three months within a year[19]. Income level was decided by the ranking among the similar age group of 5 years interval and education level was decided by their final academic degree.

### Spirometry

Spirometry was conducted by specially trained pulmonary technicians using digital computed spirometry (Sensomedic, Anaheim, CA, USA) according to American Thoracic Society (ATS) recommendations[20]. At least three maneuvers were performed and the best measures were recorded. In brief, each acceptable curve should not include any of following; an unsatisfactory start of expiration, coughing, early termination of expiration, a Valsalve maneuver, a leak, an obstructive mouth piece or an air leak. The electronically generated spirometry data were transferred via internet to the review center on the same day, where two trained nurses reviewed the test results and provided quality control feedback to the technicians according to ATS recommendation and NHANES respiratory Health Spirometry procedures Manual. The predicted forced expiratory volume in one second (FEV_1_) and forced vital capacity (FVC) were derived from the survey data of lifetime non-smoking participants with normal chest radiographs and no history of respiratory disease or symptoms[21]. Airflow limitation was defined as having an FEV_1_ to FVC ratio (FEV_1_/FVC) of less than 0.7. For estimation of FEV_1_ decline as smoking, all subjects were enrolled in analysis.

### Chest radiograph

A posterior-anterior chest radiograph was obtained during deep inspiration in a standing position with a digital radiography unit (digiRAD-PG, Sitec Medical, Gimpo, Korea). The radiographs were evaluated by at least two specialists including a pulmonologist and a radiologist using standard criteria for reporting of radiologic abnormalities[22]. Inactive TB on chest radiograph was defined as follows: the presence of discrete linear or reticular fibrotic scars, or dense nodules with distinct margins, with or without calcification, within the upper lobes[23]. Discordant findings were resolved by discussion with a second radiologist to reach a consensus and the concordance rate of the chest radiograph reading was 95.3% in this study.

### Statistical analysis

To examine the overall characteristics between participants with and without airflow limitation, chi-square test and Wilcoxon two-sample test were used. Multivariable logistic regression models were fitted to investigate the effects of inactive TB, asthma, and smoking on airflow limitation. The odds ratios (ORs) for airflow limitation were estimated, adjusting for potential confounders (age, sex, body mass index, education, and income level). To explore the interaction between inactive TB and smoking, and asthma and smoking, we added their interaction terms in the multivariable models, which already included the main effect term for inactive TB, asthma, smoking and other confounders[24,25]. Thus, we investigate whether effect of TB or asthma contributes to the risk of being with airflow limitation depending on the level of smoking or not.

To explore the relationship between levels of smoking and FEV_1_/FVC, the Generalized Additive Models for Location, Scale and Shape (GAMLSS)[26] approach proposed by Rigby were fitted for the subjects with no risk factors, with inactive TB, and with asthma, respectively. Box-Cox *t* distribution was the best fitted distribution of FEV_1_/FVC for each population, and location, scale and the skewness parameters were modeled based on the best generalized Akaike Information Criterion (GAIC). The predicted values of 50 percentiles were used to calculate corresponding smoking pack-year. The fitness of the model was checked based on the normal Q-Q plots and worm plots. Significance was defined as two-tailed p-values less than 0.05. Statistical analyses were done with SPSS for Windows (version 18.0, SPSS Inc., Chicago, IL, USA) and analysis of GAMLSS and graphs were done in R (version 3.0.2, http://www.r-project.org).

## Results

### Study population

**Fig 1:**
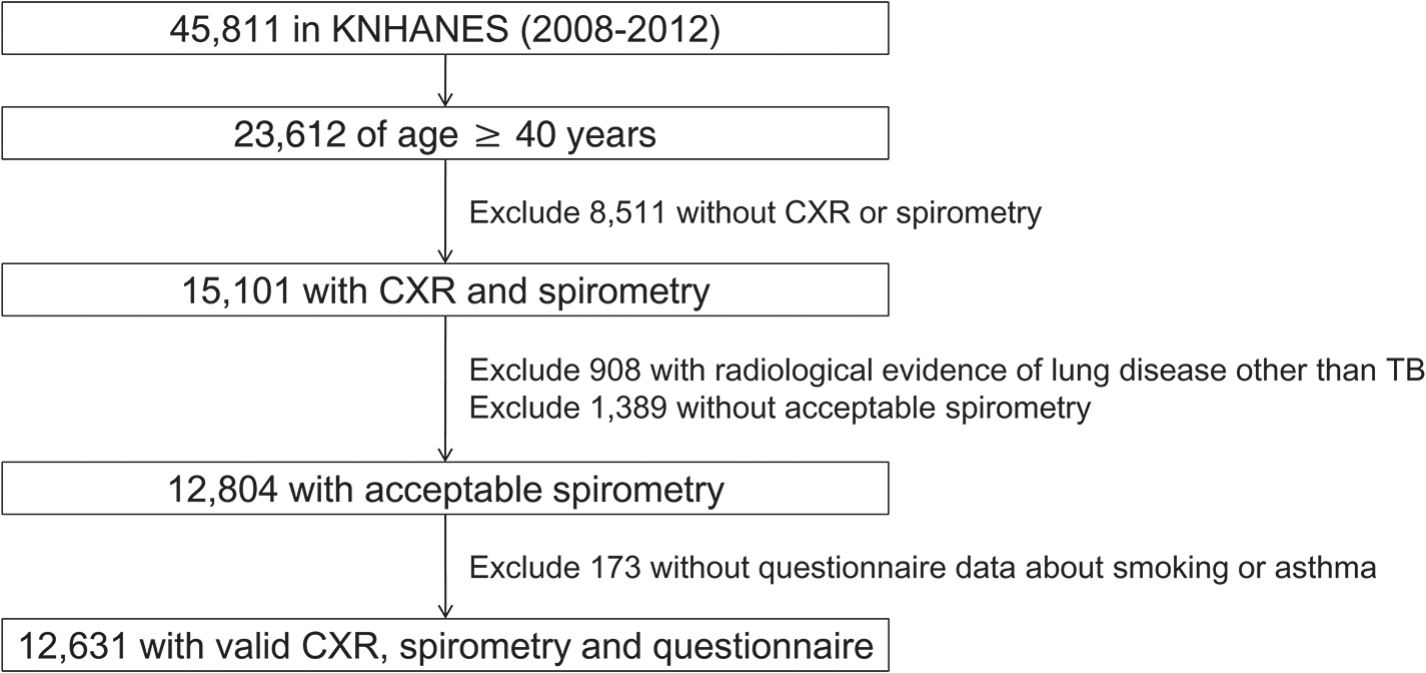
Flow diagram of study participants. KNHANES = Korean National Health and Nutrition Examination Survey.

Of 23,612 participants over 40 years old in KNHANES, 12,631 were eligible (Fig 1). Among the enrolled participants, 3,829 (30.3%) participants smoked over 10 pack-years (male, 95.1%; female, 4.9%) and 7,514 (59.5%) were never-smoker (male, 11.1%; female, 88.9%). Asthma was reported in 563 (4.5%), of whom 399 (70.9%) were diagnosed by physician and 141 (25.0%) answered that they underwent treatment for asthma. Inactive TB lesion on chest radiograph was noted in 1,048 (8.3%) participants, and 757 (6.0%) answered that they had a previous history of TB. The prevalence of airflow limitation (FEV_1_/FVC<0.7) was 12.3% (1,548/12,631), with significantly more males than females in this group (male, 72.3%, vs. female, 27.7%; *P*<0.001). The majority of those with airflow limitation had mild (FEV_1_≥80%, 747/1,548, 48.3%) or moderate (80%>FEV_1_≥50%, 738/1,548, 47.7%) airflow limitation. Among those with airflow limitation, 29.7% (460/1,548) had never smoked (Table 1).

**Table 1:** Baseline characteristics of the participants.

	All participants (n = 12,631)
Male	5,400 (42.8)
Age, yr (median[range])	55.0 (40–91)
Smoking status	
Never	7,514 (59.5)
Former	1,850 (14.6)
Current	3,267 (25.9)
Smoking history, pack-years	
Never	7,514 (59.5)
0.1−9.9	1,288 (10.2)
10−19.9	1,128 (8.9)
20−29.9	1,101 (8.7)
30−39.9	764 (6.0)
40+	836 (6.6)
Asthma	563 (4.5)
Chest radiograph	
Normal	11,583 (91.7)
Inactive TB	1,048 (8.3)
BMI, kg/m^2^ (mean±SD)	24.3 ± 3.0
Chronic bronchitis*	610 (7.3)
Wheezing (ever experienced)	1,047 (8.3)
Education level (highest degree)	
Elementary school	4,091 (32.4)
Middle school	2,013 (16.0)
High school	3,918 (31.1)
Above university	2,596 (20.6)
Pulmonary function test	
FVC, L (mean ± SD)	3.45 ± 0.84
% predicted (mean ± SD)	92.97 ± 11.68
FEV_1_,L (mean ± SD)	2.68 ± 0.67
% predicted (mean ± SD)	92.82 ± 13.32
FEV_1_/FVC (mean ± SD)	0.78 ± 0.07

Definition of abbreviations; PY pack-years, BMI body mass index, FVC forced vital capacity, FEV_1_forced expiratory volume in one second

*Cough or sputum for more than three months within a year. Proportion is calculated after excluding 4,324 participants without answer.

Values are represented as number (%), or mean ± standard deviation unless otherwise stated.

### Prevalence of airflow limitation according to risk factors

**Fig 2:**
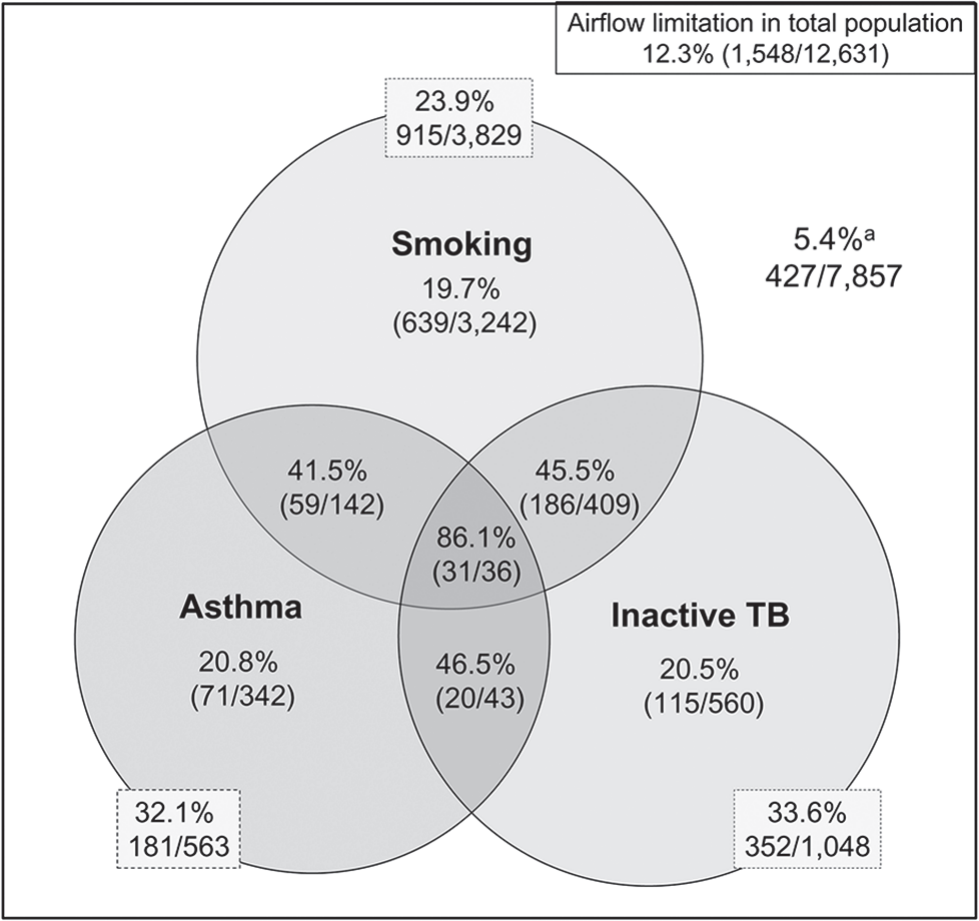
The prevalence of airflow limitation according to participant characteristics. Smoking is defined as ≥ 10 pack-years. Airflow limitation is defined as FEV_1_/FVC < 0.7. *Participants with smoking history less than 10 pack-years and without inactive TB and asthma.

The prevalence of airflow limitation in smokers of ≥ 10 pack-years, asthmatics, and those with inactive TB was 23.9% (2915/3,829, 95% confidence interval [CI] 23.2 to 24.6%,), 32.1% (181/563, 95% CI 28.3 to 36.0%), and 33.6% (352/1,048, 95% CI 30.7 to 36.5%), respectively. The prevalence was about 20%, if participants had just one risk factor (smoking 19.7%, [95% CI 18.3 to 21.1%], asthma 20.8% [95% CI 16.5 to 25.0%] and inactive TB 20.5% [95% CI 17.2 to 23.9%]). However, the prevalence increased if participants had two or more risk factors: airflow limitation was more common in smokers (≥ 10 pack years) who had a history of asthma (41.5%, 59/142, 95% CI 33.4 to 49.7%) or TB (45.5%, 186/409, 95% CI 40.6 to 50.3%). In participants with all three of these risk factors, prevalence of airflow limitation increased to 86.1% (31/36, 95% CI 74.8 to 97.4%). Meanwhile, 5.4% (427/7,857, 95% CI 4.9 to 5.9%) of the participants without any of these three risk factors had airflow limitation (Fig 2).

### Pulmonary function in smokers with asthma or inactive TB

**Fig 3:**
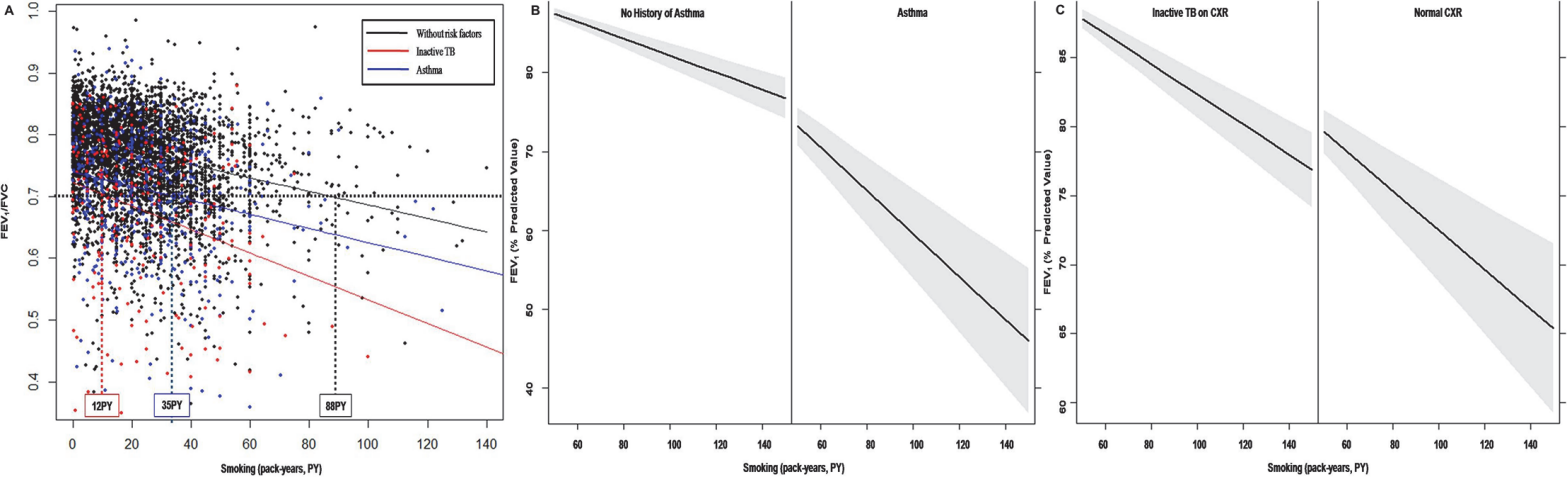
Development of airflow limitation according to smoking history and underlying disease. *(A)* Plots of FEV_1_/FVC according to smoking intensity and its 50th percentile prediction line show lower levels of smoking are required for airflow limitation in participants with asthma or inactive TB than in those without. *(B*, *C)* FEV_1_ was lower in participants with asthma or inactive TB than in those without. The gray region represents the 95% confidence interval of FEV_1_. FVC = forced vital capacity, FEV_1_ = forced expiratory volume in one second.

The FEV_1_/FVC ratio correlated inversely with extent of total smoking. Among never smokers, FEV_1_/FVC of subjects with asthma or inactive TB was lower that of those without asthma or inactive TB. The 50 percentile prediction curve of pulmonary function using GAMLSS indicated that FEV_1_/FVC of 0.7 corresponded to 88 pack-years of smoking history in those without asthma or inactive TB, but to 12 and 35 pack-years in participants with asthma and inactive TB, respectively, both of which were significantly lower than that of the nonsmokers without asthma or inactive TB (*P*<0.001, Fig 3A). FEV_1_ of asthmatics also decreased with increased smoking to a level below that of those without asthma (Fig 3B). A similar FEV_1_ trend was noted in participants with inactive TB lesions (Fig 3C).

### Association and interaction of risk factors for airflow limitation

In the unadjusted analysis, smoking, asthma, and inactive TB were associated with airflow limitation. Inactive TB had the highest OR at 4.39 (95% CI 3.81 to 5.06). After adjustment for biologic (model 1) and socioeconomic factors additionally (model 2), each of the three risk factors was still associated with airflow limitation (Table 2). In the sensitivity analysis by using definition of asthma as ‘wheezing in the last year’, it is still associated with airflow limitation; ORs were 3.01 (95% CI 2.59 to 3.49) in unadjusted model, 3.23 (95% CI 2.72 to 3.85) in model 1 and 3.20 (95% CI 2.69 to 3.81) in model 2. The fully adjusted data showed that asthma and inactive TB were equivalent to smoking histories of 69 (95% CI, 58 to 81) and 47 pack-years (95% CI 38 to 55), respectively.

**Table 2:** Risk of airflow limitation according to asthma, TB and smoking, and interactions between these risk factors.

	Airflow limitation, OR (95% CI)		
	Unadjusted	Model 1^1^	Model 2^2^
Inactive TB	4.39 (3.81–5.06)	2.44 (2.06–2.88)	2.43 (2.06–2.87)
Asthma	3.71 (3.08–4.47)	3.82 (3.06–4.77)	3.76 (3.01–4.70)
Smoking^3^	1.66 (1.60–1.72)	1.22 (1.17–1.27)	1.21 (1.17–1.25)
Inactive TB-smoking interaction		*P* = 0.07	*P* = 0.054
Asthma-smoking interaction		*P* = 0.44	

Definition of abbreviations: OR odds ratios, CI confidence interval.

Reference category; never-smokers without asthma and TB evidence on chest radiograph

^1^Model1: adjusted with age, gender, BMI, asthma, TB, and smoking.

^2^Model2: adjusted with parameters of model 1 plus education. Income was excluded in analysis due to interaction with education.

^3^Smoking; per 10 pack-years increment.

**Fig 4:**
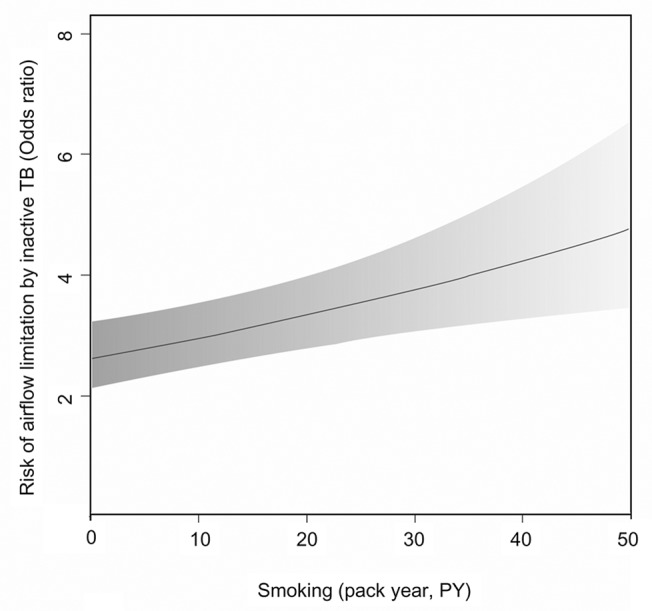
The impact of inactive TB on airflow limitation according to smoking history. The gray region represents the 95% confidence interval of the odds ratios. PY = pack-years.

We observed a potential two-way interaction between inactive TB and smoking on airflow limitation (*P* = 0.054), but no asthma-smoking interaction was found. The presence of a significant interaction indicates that the effect of one predictor variable on the response variable is different at different values of the other predictor variable. Significance of the coefficient of estimate for interaction terms indicated that TB or asthma effect on airflow limitation differs significantly with their smoking level (pack-year). Although the inactive TB-smoking interaction did not reach statistical significance, the risk of airflow limitation due to inactive TB increased clearly as the amount of lifetime smoking increased: the OR for airflow limitation by inactive TB was 2.77 (95% CI, 2.24 to 3.43) in never-smokers, but it increased to 3.13 (95% CI 2.61 to 3.76) in smokers of 10 pack-years and 3.54 (95% CI 2.96 to 4.24) in those of 20 pack-years (Fig 4).

## Discussion

In this study, we showed that in addition to smoking, TB scar and asthma history had a great impact on the development of airflow limitation. These factors increased the prevalence of airflow limitation independently, and the prevalence increased markedly if the participants had two or more risk factors. As a result, participants with asthma or inactive TB could develop airflow limitation with a lower amount of smoking than participants without these risk factors. We also found a potential interaction between inactive TB and smoking for airflow limitation that was of marginal statistical significance.

Several factors including smoking, asthma, TB history, aging and biomass fuel are well-known factors associated with irreversible airflow limitation, which is the main characteristic of COPD. Control of these factors is the most effective way to manage COPD. Although they are reported as separate risk factors, many patients have two or more of these associated factors; therefore, it is important to study their combined effects. A lower level of smoking was associated with airflow limitation (FEV_1_/FVC < 0.7), an important criteria of COPD, in participants with history of asthma or inactive TB. Prediction curve of FEV_1_/FVC suggested that 88 pack-years of smoking was necessary for the development of airflow limitation in individuals without history of asthma or TB, but only 12 or 35 pack-year smoking might be enough in individuals with history of asthma or TB, respectively. As a result, the prevalence of airflow limitation was increased in participants with these risk factors. About half of the smokers with a history of TB or asthma had airflow limitation. Furthermore, the probability of airflow limitation was more than 80% in smokers with both asthma and inactive TB lesions compared to about 40% in smokers with history of either asthma or inactive TB lesions. The associations with airflow limitation in middle age indicated that inactive TB was equivalent to 47 pack-years and asthma was 69 pack-years. FEV_1_ of subjects with asthma or inactive TB also decreased with increased smoking to a level below that of subjects without those associated factors, and that trend was notified as the smoking amount, irrespective of FEV_1_ level[27]. These data could form the basis for the development of comprehensive healthcare strategies. It is clearly important to discourage smoking, but our data suggest an additional focus on patients with a history of chronic respiratory disease, particularly if we accept that anti-smoking campaigns will not influence all people.

A potential interaction (multiplicative effect) between smoking and inactive TB in the development of airflow limitation was identified, with marginal significance (*P* = 0.054). Thus, the risk of airflow limitation from inactive TB might increase more than the individual smoking and inactive TB associations combined. A history of TB affects lung function by developing obstructive dysfunction, and the effects can differ according to site and extent of involvement[10–12]. Some subjects with TB history can have a borderline obstructive pattern in pulmonary function, although not below the normal level. In these people, airflow limitation due to smoking can develop more readily than in the people without TB history. A previous report also reported that the association between inactive TB and airflow limitation was stronger in smokers than in never-smokers[10]. An interaction between asthma and smoking in airflow limitation was not observed in our study. A previous study did identify an interaction between asthma and smoking, but only in those with atopic sensitization[28].

Although 10 pack-years smoking is enough to increase the risk of COPD[17,18],our results indicated that people without a history of respiratory disease would have airflow limitation after an average of about 88 pack-years of smoking. Lung function falls gradually over a lifetime due to smoking[27], but only a small proportion will develop clinically relevant disease even after extended period of smoking[29,30].Therefore, high levels of smoking could be necessary to cause airflow limitation in most people. Badgett and colleagues found that the only useful history to predict airflow limitation caused by smoking was self-reported history of chronic obstructive airway disease and smoking more than 70 pack-years[31],which accords with our report. In another report, 40 pack-years was suggested to be the most appropriate cut-off for a diagnosis of COPD[32].

Chronic asthma can carry a greater risk than smoking to have some characteristics feature of COPD[33]. Asthmatics were more likely to develop chronic bronchitis or emphysema with more than 10 times probability than those without asthma even after adjustment for confounding factors[9]. Early-onset asthma without remission is an important predictor for fixed airflow limitation[28], because the reduced lung function cannot recover to reference levels during and after progression into adulthood[15]. Several longitudinal studies also reported that a decline in FEV_1_ was greater in asthmatics, with smoking significantly accelerating this decline[14,34]. Fixed airflow limitation can arise from airway remodeling associated with longstanding asthma. Regular inhaled corticosteroid (ICS) treatment has shown some potential for prevention of airway remodeling, but this effect has only been shown in relatively short-term trials using high doses, sometimes at doses higher than used in clinical practice[35,36]. By contrast, ICS at a standard dose failed to show a definite improvement in airway remodeling in long-term studies[37–39]. The contribution of asthma for COPD is likely to increase in importance, particularly in view of asthma and increasing longevity worldwide.

There are several limitations to this study. First, the extent of TB lesions on chest radiograph or asthma severity could not be evaluated due to the limited information available. The immune response to TB can cause airway inflammation followed by airway fibrosis and bronchial stenosis[40]; therefore, more extensive TB lesions could cause more severe inflammation and lung function decline, which could increase the susceptibility of the airways to smoking. Patients with severe and uncontrolled asthma can also have airflow limitation with high probability. Second, asthma was self-reported in the study, which could potentially result in misdiagnosis of similar clinical manifestations such as COPD or chronic bronchitis. Previous cross-sectional studies have shown a large overlap of up to 30% between people who have a clinical diagnosis of COPD and asthma[41]. However, the definition of asthma in population based study is not easy; therefore many epidemiologic studies used similar method based on questionnaire and previous study showed self-reported asthma could be also reliable[19]. In our study, 80% of asthmatics were diagnosed by physicians, and the agreement between self-reported asthma and physician diagnosed asthma was good with κ value of 0.823. Third, post-bronchodilator spirometry was not performed in KNHANES. Since it is necessary to exclude reversible airflow limitation for the diagnosis of COPD, this could lead to an overestimate of the risk of airflow limitation attributable to asthma. Fourth, some potential risk factors such as biomass fuel or second hand smoking were not included in analysis. Recently, indoor biomass cooking is extremely rare in Korea and it is not surveyed. Only second hand smoking itself does not affect the risk of COPD, and intensity and duration should be considered[42], which was not available in our survey. Despite these limitations, the message from our study is still valid. The trend of lung function decline due to smoking was also noted in asthmatics, and participants with TB lesions exhibited airflow limitation associated with a much lower level of smoking.

The data suggest that in people with chronic respiratory diseases such as TB or asthma, lower levels of smoking are required to develop clinically significant airflow limitation. COPD, TB and asthma are all major public health problems. To prevent the development of secondary disease, it is important to manage not only each of these diseases, but also the smoking after the treatment. The results from this population-based study will be useful in indicating the direction of public health strategy, especially anti-smoking campaigns.

## Supporting Information

S1 Table Sensitivity analysis including chronic respiratory symptoms. (DOCX)
