# Supplementary material for: The Impact of Smoking on Airflow Limitation in Subjects with History of Asthma and Inactive Tuberculosis
Source: PLoS One. 2015 Apr 27;10(4):e0125020. doi: 10.1371/journal.pone.0125020 (PMC4411068; doi:10.1371/journal.pone.0125020)
Supplement: S1 Table — (DOCX) [file pone.0125020.s001.docx]

**S1 Table. Sensitivity analysis including chronic respiratory symptoms.** The range of fluctuation of odds ratios according to additional variable (chronic respiratory symptoms) is less than 15%.

|  | **Airflow limitation, OR (95% CI)** | | |
| --- | --- | --- | --- |
|  | **Unadjusted** | **Model 1^1^** | **Model 2^2^** |
| Inactive TB | 4.39 (3.81–5.06) | 2.44 (2.06–2.88) | 2.43 (2.06–2.87) |
|  | 4.39 (3.81–5.06) | 2.44 (2.06–2.88) | 2.43(2.05–2.87) |
| Asthma | 3.71 (3.08–4.47) | 3.82 (3.06–4.77) | 3.76 (3.01–4.70) |
|  | 3.71 (3.08–4.47) | 3.76 (3.00–4.70) | 3.70(2.96–4.64) |
| Smoking^3^ | 1.66 (1.60–1.72) | 1.22 (1.17–1.27) | 1.21 (1.17–1.25) |
|  | 1.66 (1.60–1.72) | 1.22 (1.18–1.26) | 1.21(1.17–1.25) |
| Inactive TB-smoking interaction |  | *P*= 0.07 | *P*= 0.054 |
|  |  | *P=0.09* | *P=0.78* |
| Asthma-smoking interaction |  | *P* = 0.44 |  |
|  |  | *P=0.39* | *P=0.37* |

Upper rows unshaded : without chronic respiratory symptoms

Lower rows shaded : with chronic respiratory symptoms

Definition of abbreviations: OR odds ratios, CI confidence interval.

Reference category; never-smokers without asthma and TB evidence on chest radiograph

^1^Model 1: adjusted with age, gender, BMI, asthma, TB, and smoking.

^2^Model 2: adjusted with parameters of model 1 plus education. Income was excluded in analysis due to interaction with education.

^3^Smoking; per 10 pack-years increment.
